# Human rights violations in organ procurement practice in China

**DOI:** 10.1186/s12910-017-0169-x

**Published:** 2017-02-08

**Authors:** Norbert W. Paul, Arthur Caplan, Michael E. Shapiro, Charl Els, Kirk C. Allison, Huige Li

**Affiliations:** 1 Institute for History, Philosophy and Ethics of Medicine, Johannes Gutenberg University Medical Center, Mainz, Germany; 2 Division of Medical Ethics, School of Medicine, New York University, New York, NY USA; 3 Department of Surgery, Rutgers - New Jersey Medical School, Newark, USA; 4 Department of Psychiatry, University of Alberta, Edmonton, Canada; 5 School of Public Health, University of Minnesota, Minneapolis, USA; 6 Department of Pharmacology, Johannes Gutenberg University Medical Center, Obere Zahlbacher Strasse 67, 55131 Mainz, Germany

**Keywords:** Organ procurement, Prisoners, Human rights, Medical ethics, China

## Abstract

**Background:**

Over 90% of the organs transplanted in China before 2010 were procured from prisoners. Although Chinese officials announced in December 2014 that the country would completely cease using organs harvested from prisoners, no regulatory adjustments or changes in China’s organ donation laws followed. As a result, the use of prisoner organs remains legal in China if consent is obtained.

**Discussion:**

We have collected and analysed available evidence on human rights violations in the organ procurement practice in China. We demonstrate that the practice not only violates international ethics standards, it is also associated with a large scale neglect of fundamental human rights. This includes organ procurement without consent from prisoners or their families as well as procurement of organs from incompletely executed, still-living prisoners. The human rights critique of these practices will also address the specific situatedness of prisoners, often conditioned and traumatized by a cascade of human rights abuses in judicial structures.

**Conclusion:**

To end the unethical practice and the abuse associated with it, we suggest to inextricably bind the use of human organs procured in the Chinese transplant system to enacting Chinese legislation prohibiting the use of organs from executed prisoners and making explicit rules for law enforcement. Other than that, the international community must cease to abet the continuation of the present system by demanding an authoritative ban on the use of organs from executed Chinese prisoners.

**Electronic supplementary material:**

The online version of this article (doi:10.1186/s12910-017-0169-x) contains supplementary material, which is available to authorized users.

## Background

Before a pilot organ donation program was introduced in 2010, at least 90% of the transplanted organs in China were procured from prisoners [1]. China is the only country in the world that systematically uses organs from prisoners for transplantation [2]. After decades of denial, China finally admitted the practice in 2005 [3, 4]. However, organ procurement from executed prisoners continued.

In December 2014, the Chair of the China Organ Donation and Transplant Committee and former Vice-Minister of Health, Huang Jiefu, announced that the country would completely cease the use of prisoner organs for transplantation in China after 2015. Surprisingly, this announcement has not been followed by any changes to China’s organ donation laws or regulations (see website of PRC National Health and Family Planning Commission: http://en.nhfpc.gov.cn/regulations.html). The use of prisoner organs remains legal, if so-called consent is obtained from the prisoners [5, 6]. Such ‘consents’, however, even those actually signed by the prisoners, are not accepted by international organisations such as The Transplantation Society (TTS) [7]. Because of the restrictions on liberty in a prison environment, it is unlikely that prisoners are truly free to make independent decisions and thus an autonomous informed consent for donation cannot be obtained [7].

Organ harvesting from executed prisoners violates international principles of medical ethics [8]. The compromised autonomy of all prisoners as an ethical restriction regarding the process of informed consent had historically been derived from a thorough analysis of crimes against humanity performed by physicians during the German Nazi regime mostly on prisoners in German concentration camps. In Asia, crimes against humanity performed during World War II were shamefully not prosecuted in a similar way as the Nuremberg Military Tribunals. The vivisections and experimental atrocities performed by Japanese biological warfare Unit 731 against occupied Chinese and prisoners of war in Manchuria were not raised, addressed nor prosecuted during the Tokyo Trials [9, 10].

The unethical practice of organ procurement from executed prisoners in China has lasted for decades [5]. Moreover, this practice is associated with large scale abuse and severe human rights violations. In part, the transgression of ethical boundaries in the case of organ harvesting is triggered by an ever growing local and global demand for transplantable organs associated with the emergence of a global (black) market [11–13]. This heavily impacts human rights, ethics and justice far beyond ideas calling for regulated markets for organs [14].

In a prior publication, we explored the historical development and status of organ procurement from death-row prisoners in China [5]. In the present article, we more closely examine transgressions of human rights (mainly focusing on legal considerations of human rights), contradictory frames offered in justification, conditions claimed for consent of this most vulnerable of populations, issues of brain death criteria in the context of China, and several specific examples.

## Organ procurement from executed prisoners without consent

From the 1970s, organs were simply taken from executed prisoners in China without asking for the permission of the prisoners or their families. In 1984, the first “Interim Rules on Using the Body or Organs of Executed Criminals” [15] (also translated as “Provisional Regulation on the Use of Dead Bodies or Organs from Condemned Criminals” [16]) officially allowed, for the first time, organ harvesting from executed prisoners, under the condition that either the body is not claimed; or the prisoner volunteers for organ donation; or the family consents.

As prescribed by the 1984 Rules, “the use of the dead bodies or organs from condemned criminals must be kept strictly confidential. […] the operation vehicles from medical institutions can be allowed entry into the execution grounds to remove organs, but vehicles with the logo of medical institutions are not to be used, and white clinic garments are not to be worn. The execution ground should be guarded against before the operation is completed. After the dead bodies are used, the crematory shall assist the [medical] units in timely cremation” [16]. In many cases, the families receive only the cremains without being informed what has happened with the body of their loved ones.

Recently, it has been repeatedly admitted by Chinese transplant officials that organs from executed prisoners had been procured without consent [17–19]. For example, a Chinese medical official admitted in 2013 that “previously, authorities used executed criminals’ organs without their consent, while permission has been required in recent years” [17]. In 2014, when Hong Kong newspaper Mingpao asked the question whether prisoners and their families are informed and consent obtained before organ procurement, Huang Jiefu admitted that “we have not been able to achieve this, but we will soon” [18]. In 2015, Huang Jiefu admitted that the problem with China’s organ transplantation system was that the laws were not obeyed: “Although the law provides that the use of prisoner organs must be from voluntary donation, but there are still loopholes in the law enforcement” [19]. All of these statements strongly suggest that organs were procured in China largely without consent of the prisoners or their families.

Although organ procurement without consent is illegal according to Chinese laws, the practice appears to be tolerated by the authorities in China.

## Organ procurement from incompletely executed prisoners

At a European Parliament hearing on 29 January 2013, Enver Tohti testified that he was ordered in 1995 to harvest organs from an incompletely executed, still-living prisoner in China [20]. More details were revealed in his interviews with ABC [21], BBC [22] and journalist Ethan Gutmann [23, 24]. When Enver Tohti got the body of the ‘executed’ prisoner, he noticed, however, that the prisoner was not dead. “The gunshot, gun wound was on his right chest. So, I guess that was deliberately to make this prisoner not die immediately to allow some time for us to remove that organ when he is still alive” [21]. When Tohti kept making attempts to follow normal procedure – sterilize, minimal exposure, sketch the cut - the chief surgeon told him to hurry up. “No anesthesia, no life support”, “we are working against time”. Finally, Tohti extracted the liver and the kidneys from the still-living prisoner [23, 24].

Unfortunately, this was not the only case reported. An early case has been documented in the book “China’s eyes” [25]. In 1978, Zhong Haiyuan, a schoolteacher from the Jiangxi Province, was sentenced to death for her “counterrevolutionary” thoughts. The execution was performed by three officers from the People’s Armed Police Forces on April 30, 1978. Two officers fixed Zhong while the third officer put the gun against her back on the right side and fired the bullet [25]. Years later, one of the officers told the books’ author that the order was not to kill Zhong immediately. “The kidneys must be harvested before she dies”, because the army doctors wanted high quality kidneys, “kidneys from a living person” [25].

More recently, Mr. Wang, who currently lives in Canada, revealed that he was once a member of a team extracting organs from a still-living person. The incident happened in the 1990s when Wang was an intern doctor at the urology department of the Shenyang Military General Hospital in Liaoning Province [26].

In March 2015, Jiang Yanyong told to Hong Kong journalists that corruption, illegal transplantation and organ trade were common in military hospitals [27, 28]. Jiang elaborated on the case of Li Shiyong, director of the Department of General Surgery of the Beijing Military General Hospital. With no prior experience in transplantation and without asking for permission of the hospital director, Li founded a liver transplantation center in 2005 and appointed himself as the director. Because Li had found ways to obtain donor livers, he could serve as the director (see Additional file 1: Table S1).

In the same TV interview, Jiang also revealed that many of the prisoners used for organ harvesting were shot but not completely killed [27, 28]. The purpose was for organ harvesting while the prisoners were still alive in order to keep the warm ischemic time of the sourced organs as short as possible (see Additional file 1: Table S1).

Jiang as a source is, we contend, credible based on his personal story within the Chinese medical system. He was a chief physician of the 301 Military Hospital (People’s Liberation Army General Hospital) in Beijing where he witnessed the results of the trauma inflicted on the students during the Tiananmen Square Massacre of 1989. Jiang was also the person who publicized the cover-up of the Severe Acute Respiratory Syndrome (SARS) epidemic by the Chinese government in 2003.

Unfortunately, the strength of these examples is constrained by the fact that the number of executions and the detailed techniques used are state secrets in China; empirical data cannot be generated. There are no statistics available on the incidence of the practice of incomplete executions in China. A systematic international investigation into this issue is needed in the interest of (overdue) justice for the victims.

## The medical and ethical function of brain death and its implementation in China

In most Western cultures, treating a person only as a mere means to an end of another is a challenge to core concepts of human dignity as Immanuel Kant and other 18th century philosophers argued [29]. Even from a contemporary perspective, ethicists have always argued that transferring organs to another person either from a living donor or from post mortem procured organs must be contextualized in a way that a) human dignity, b) autonomy and c) social justifiability in the light of shared values are not endangered [30, 31].

Here, human dignity is understood to be an applied concept. Based on the notion of phenomenological dignity it is cognition that enables humans to identify themselves and to perceive others as human beings which are to be treated with the same respect (dignity) that one would expect for oneself. This concept is fostered by a reflexive mode of dignity in which the constant (re-)evaluation and adjustment of actions is put in place by relating both, intentions and consequences to commonly accepted values, like those which constitute our understanding of human rights. Finally, the mutual appreciation of humans together with a value based evaluation and adjustment of interaction turn dignity into a relational concept which ought to be the guiding principle of human coexistence. Especially the relational dimension of dignity is addressed in discourses on brain death when it comes to evaluations of those values, moral concepts and needs which need to be reflected for both, donors and recipients of organs [32].

Only this clarification could lead to a situation in which the concept of brain death established the medical basis and justification to switch from life-sustaining care setting to considering a deceased person as a cadaver organ donor. Ethically, brain death is the fine line on which dignity of a living and acting person changes into the dignity of a deceased person, and respecting autonomy of a living person shifts over into respecting an advanced directive or declared will to donate organs. In this context, the justifiability of organ procurement is derived from the fact that the brain-dead person can donate organs without suffering from vital consequences caused by organ removal (e.g. pain and death), since he or she is already dead. Intensive care is provided only to protect the organs of the deceased.

It is this integration of medical and ethical functions of brain death which makes organ procurement a widely accepted practice in Western culture. This is especially true because of the potential for other procurement strategies, such as procurement from non-heart-beating donors (Donation after Circulatory Death - DCD), which require careful, detailed and transparent protocols, in order to assure the avoidance of even the potential for conflict of interest regarding treatment, and verification of the death of the donor [33, 34]. Circulatory death is, if not clearly defined as the irreversible cessation of cardiovascular circulation, potentially reversible. Furthermore, it can be prognostically highly dependent on a number of arbitrary, concomitant circumstances (time, temperature, cause), thus requiring strict protocols, including electrocardiography and blood pressure monitoring to assure death has occurred, and is permanent. DCD donation may also lead to decreased quality of recovered organs, because of prolonged ischemia, or reduced number of organs that can be procured. It thus has to be seen as a procedure only applicable in an environment with reliable clinical standard operating procedures (SOPs) which have to be implemented in an evidence-based mode by well-trained medical personnel [34, 35].

However, there is yet no brain death legislation in China and circulatory death is the legal standard despite the absence of evidence-based and reliable SOPs [36]. China lacks any state issued official guidelines to diagnose brain death.

In 2003, the Ministry of Health drafted “Brain Death Determination Criteria” and “Brain Death Determination Technical Specifications” (comment drafts). In 2009, the Ministry of Health revised the two documents. In March 2012, the National Health and Family Planning Commission (NHFPC, the former Ministry of Health) assigned Xuanwu Hospital of Capital Medical University as the Brain Injury Quality Control Evaluation Center. In 2013, the Center further revised and combined the two documents into “Brain Death Determination Criteria and Technical Specification (Adult Quality Control Version)” [37]. These Criteria and Technical Specifications represent a *suggested* medical standard; it is not a standard procedure, a mandatory guideline for medical practitioners or an administrative regulation. Above all, the standard is not legally binding. As stated in the Editor’s Note accompanying its publication, “the Center has revised and improved the above-mentioned documents on the basis of 10 years of clinical practice and research on brain death determination, and hopes that the new document serves as the medical standard to promote the brain death determination in our country to develop orderly and normatively” [37].

**Fig. 1:**
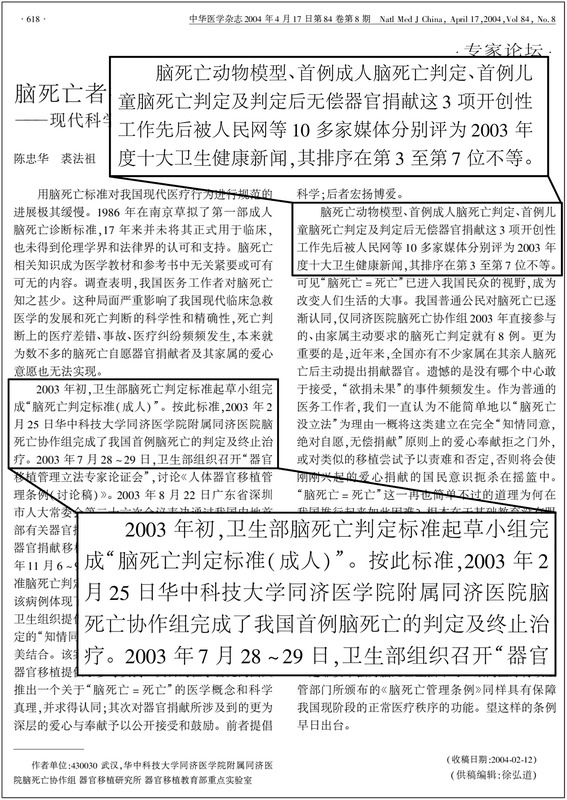
The first brain death determination in China. Shown is an Expert Opinion paper published in National Medical Journal of China (Zhonghua Yi Xue Za Zhi), a top medical journal in China, by Chen & Qiu [40]. Text in left box: ‘At the beginning of 2003, the drafting group of the Ministry of Health completed the “Brain Death Determination Criteria (for adults)”. According to these criteria, the Brain Death Coordinating Group of the Tongji Hospital at the Tongji Medical College, Huazhong University of Science and Technology, completed the first brain death determination and treatment cessation in China on 25 February 2003’. Text in right box: ‘The animal model of brain death, the first brain death determination in adults, and the first brain death in children followed by unpaid organ donation – these three pioneering works were nominated by 10 major Chinese media to be among the top ten medical news of 2003, ranking from 3 to 7, respectively’

The first documented brain death diagnosis in China was performed on 25 February 2003, in Wuhan, Hubei Province [38]. The brain death determination was carried out according to the published 2003 draft. In November 2003, the kidneys of a boy diagnosed as brain dead were used for transplantation with the consent of the parents [39]. Both events were considered breakthroughs in Chinese transplant medicine. Both were nominated by major Chinese media to be among the top 10 medical stories of 2003 [40] (Fig. 1). Since then, there have been reported increasing numbers of organ donations after brain death, although “it is illegal to take organs from the brain-dead for transplant purposes” in China, as acknowledged by China Daily [41].

Even though no brain death legislation has been effectuated in China, the Chinese Classification of Deceased Organ Donation has been recently formulated as follows: “China Category I: Organ donation after Brain Death; China Category II: Organ Donation after Circulatory Death; China Category III: Organ Donation after Brain Death followed by Circulatory Death” [36]. The ambiguity of this classification together with the absence of SOPs, guidelines, regulations and legislation leads to a situation in which legal, medical and ethical uncertainty continues.

## Execution by organ explantation?

**Fig. 2:**
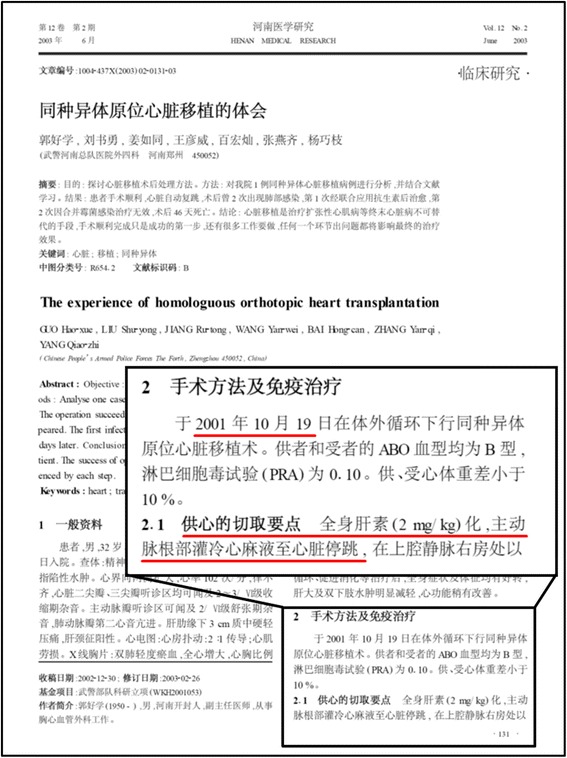
Questionable heart explantation. The operation was performed on 19 October 2001 in the hospital of the People’s Armed Police Forces in Zhengzhou City, Henan Province. That was two years before the first documented brain death determination was performed in China. Underlined text: ‘Major points of donor heart removal: systemic heparinization (2 mg/kg); delivery of cold cardioplegia to the heart through the aortic root until the heart stopped beating’. The first page of the publication by Guo et al. in the medical journal Henan Medical Research is shown [42]

The medical journal Henan Medical Research published a research paper titled “The experience of homologuous orthotopic heart transplantation” (Fig. 2). The operation was allegedly performed in a hospital of the People’s Armed Police Force in 2001 and the paper published in 2003 [42]. The 32-year-old male recipient died from infection 46 days after the heart transplantation [42].

In the section 2.1 of this research paper, the “major points of donor heart removal” included: “systemic heparinization (2 mg/kg); delivery of cold cardioplegia to the heart through the aortic root until the heart stopped beating; cut of the superior vena cava at 4 cm above right atrium …” (Fig. 2). Besides blood type and heart weight, no other information about the donor was provided in the paper.

The fact that systemic heparinization was performed and heart beating was stopped by cold cardioplegia implies that the blood was circulating and the heart was functional before the explantation procedure. Logically, one of the following two scenarios are applicable to describe the heart explantation: (i) the donor was a brain death patient and a brain death diagnosis was performed as it would have in most confirmatory brain death diagnoses based on neurological standard operating procedures (e.g. in the EUROTRANSPLANT region); (ii) the donor was not a brain death patient and the cardiac death was induced by the cold cardioplegia delivered by the medical professionals.

In this context, it is important to re-emphasize that there is no brain death legislation in China and circulatory death is still the legal standard in China [36]. As mentioned above, the first brain death determination in China was performed in 2003 (Fig. 1) [38]. The heart transplantation in this publication, however, was performed in 2001 and the concept of “brain death” is not mentioned. This therefore raises the question as to whether the donor was brain dead, especially given that the paper was published at a time where the diagnostic procedure alone would have captured the attention of the Chinese medical community and would have contributed positively to the scientific impact of the paper. It is thus reasonable to assume that the delivery of cold cardioplegia served the purpose of execution and explantation at the same time.

## Human rights: grounding and violations

On 26 June 1945 in San Francisco, the Republic of China (ROC), first in suffering Axis aggression, was also first to sign the United Nations Charter and Statute for the International Court of Justice, which entered into force on 24 October, establishing the United Nations. The latter date followed the Double Tenth Agreement (10/10/1945) between Chaing Kai-Shek and Mao Zedong wherein the Chinese Communist Party (CCP) acknowledged the Republic’s Kuomintang government as legitimate and the Kuomintang the CCP as a legitimate opposition party. Full scale civil war resumed 26 June 1946. Before Mao proclaimed the People’s Republic of China on 1 October 1949, the ROC endorsed the Universal Declaration of Human Rights (10 December 1948). The mainland People’s Republic was not a party to UN human rights instruments before 1971 but reaffirmed the UN Charter and UDHR after the Republic of China was unseated. The PRC is now party to at least 17 international instruments [43].

The United Nations Office of High Commissioner for Human Rights characterizes universal human rights as “interrelated, interdependent and indivisible” often expressed in treaties, customary international law (i.e. implicit norms) or general principles. Documents comprise moral declarations, which implicitly presuppose a concept of human flourishing, and derivative binding treaties (covenants, conventions, protocols). The troika of the 1948 UDHR and the complementary international covenants on Economic, Social and Cultural Rights (ICESCR) and Civil and Political Rights (ICCPR), respectively opened for signature in parallel in 1966, comprise the so-called ‘Bill of Human Rights’, with others building upon them.

China is a full party to the ICESCR (signed 1997, ratified 2001); the Convention against Torture and Other Cruel, Inhuman or Degrading Treatment or Punishment (CAT, ratified 1988); the Convention on the Prevention and Punishment of the Crime of Genocide (signed 1949, ratified 1983); and the United Nations Convention against Transnational Crime (ratified 2003) whose concerns include organ trafficking (albeit patients rather than prisoner organs have comprised the transnational element in China).

China also signed, but has not ratified, the ICCPR in October 1998. While formally not bound by ICCPR provisions until ratification or accession, China is obliged not to defeat its general purpose. Per the Foreign Ministry at the time, China’s 1998 signature “demonstrates its firm determination to promote and protect human rights” as well as concretely commemorating the UDHR’s 50th anniversary, acknowledging a coherence bridging moral and legal rights. It also emphasized flexibility in prioritization: “the principle of the universality of human rights must be respected, but the specific conditions of each country must also be taken into consideration in observing this principle,” highlighting economic development successes against poverty. The tone markedly contrasts more recent ideological tightening contra “Western ideas”, expressly against universal values of human rights, parallel to an anticorruption campaign short on procedural protections [44].

China domestically has circulated “alternatively phrased” translations of signed human rights conventions rather than the UN’s official Chinese text, according to one source providing an interpretive buffer while also imprisoning those who ask for the ICCPR to be ratified [45]. Legally binding, however, are the official UN language versions, not CCP state paraphrases.

Human rights violations ending in organ harvesting from prisoners are, like human rights, also interrelated and interdependent. Violation cascades – one violation increasing probability of another given the substantive interrelationship of rights - increase vulnerability cumulatively whether in a specific person or in a targeted population. Rights violations via detention schemes and a death penalty regime subject to manipulation with lack of sufficient representation or appeal [46] precondition the subsequent act of forced organ extraction: risk accumulates to a specific subject and identifiable population. In this sense, the implication of the title of this paper is extensive and systemic, not simply perioperative. De jure and de facto factors, frequently in violation of international human rights standards, lead to and condition the supply of prisoners being solicited before execution or simply exploited in the first instance.

Specifically concerning the death penalty, variability includes bringing charges across 46 potentially capital offenses; death sentences and executions waxing and waning during ‘strike hard’ campaigns; and executions increasing before the new year. Furthermore, judges in China are not unaware that executees are the mainstay of transplantation. Beyond the design of preserving public order and social control through execution is added the exogenous influence of medical demand for execution as the chief gateway to a social good (organs for transplantation), at times indirectly abetted by the international community (e.g. The Transplantation Society’s policy supporting providing training to Chinese transplant surgeons under the banner of influencing eventual reform while simultaneously expanding capacity, as in the TTS Ethics Committee Letter of 6 November 2006 to TTS members [47]). It is notable that the initial 1984 Rules officially allowing organ harvesting from executed prisoners cited the initiative of medical personnel, not the state, as seeking to exploit this context for organ sourcing [16].

The practice of organ procurement in China that we have described violates freedom from torture and other cruel, inhuman or degrading treatment or punishment (UDHR, CAT, ICCPR Art. 7). Nowhere is an individual as subject to state power than in prison, and nowhere in prison than when awaiting execution. However, it is the contiguous context, not merely local ‘choice’, that is proper object of the human rights critique, exposing the step-wise cumulative vulnerability of prisoners at risk of being exploited in and through execution. This is aided and abetted by medical demand by an occupation that first pushed for exploiting execution for organ harvesting, and by a citizen population willing to rely upon, benefit from, and exploit the bound population - evident from ongoing reticence to participate in voluntary donor registration, yet seeking transplantation surgeries.

In general, an alibi of system reform has been countenanced too long. After over a decade of reform claims and the redefinition of the status of prisoners, the ethical outcome respecting human rights in China, here conforming with international medical ethical standards, is categorical cessation of organ sourcing from prisoners. Practically, this would remove the perverse incentives shared and relied upon by medical and judicial establishments, and by the general population. Given the nature of the violations and delay, justification of gradually realizing an ethical practice fails. This recognition should hold for actors and institutions of influence outside of China now in possession of over a decade of knowledge. While human rights violations in China span systemic structural preconditions, augmented by political whim, the most proximate point of intervention still lies with the medical community and professional societies. Admitting the failure of gradualism, and increasing, rather than decreasing, professional sanctions, may more quickly realize the intent: Cessation of prisoner organ sourcing generally; reducing perverse incentives in death penalty demand (moral hazard); and confronting the general population with two ethical alternatives: supporting or declining voluntary organ transplantation as a system, while bearing the cost of either choice.

## Conclusion

The unethical practice of organ procurement from executed prisoners in China is associated with a large scale of abuse and a cascade of severe human rights violations, including, we contend, organ explantation from still-alive human beings, and, upstream, conditioning the supply of prisoners exploited per se or then solicited to ‘freely’ offer organs as atonement for real or supposed crimes. Those involved in organ harvesting from still-alive prisoners must be prosecuted. The unethical practice of lethally procuring vital organs from the living must be prevented by a law prohibiting use of prisoner organs generally, supporting change in the practical legal, medical and popular culture surrounding transplantation in China. Finally, greater influence may be exerted by international institutions through change of strategy.

## Additional file

Additional file 1:
**Table S1.** Jiang Yanyong Interview in March 2015. This additional file provides further information on Jiang Yanyong’s statements (including English translation) in his interview by Hong Kong journalists. (PDF 439 kb)

## Abbreviations

CAT: Convention against torture and other cruel, inhuman or degrading treatment or punishment
CCP: Chinese Communist Party
DCD: Donation after circulatory death
ICCPR: International covenant on civil and political rights
ICESCR: International covenant on economic social and cultural rights
PRC: People’s Republic of China
ROC: Republic of China
SARS: Severe acute respiratory syndrome
SOP: Standard operating procedure
TTS: The Transplantation Society
UDHR: Universal declaration of human rights
