# Supplementary material for: Human rights violations in organ procurement practice in China
Source: BMC Med Ethics. 2017 Feb 8;18:11. doi: 10.1186/s12910-017-0169-x (PMC5299785; doi:10.1186/s12910-017-0169-x)
Supplement: Additional file 1: — Table S1. Jiang Yanyong Interview in March 2015. This additional file provides further information on Jiang Yanyong’s statements (including English translation) in his interview by Hong Kong journalists. (PDF 439 kb) [file 12910_2017_169_MOESM1_ESM.pdf]

Table S1. Jiang Yanyong Interview in March 2015

Reference	Original Text in Chinese	English translation
**Ref. 27**	**2015 年 03 月 07 日 苹果日报**<br>**蒋彦永指出**，内地的肝移植源全部来自被处以极刑的死因，包括 301 医院、北京军区总医等都设有「器官移植中心」，这些部门主要是做器官移植和买卖等违法勾当，经济效益很高，也是医院和医护人员灰色收入的主要来源。为了能弄到器官，他们和公检法等串通，只要有死囚枪伏法，就派车到刑场接尸。**有的犯人一枪还未被打死，就被拉回医院手术台摘除器官，然后向患者移植。其手法惨无人道，令人发指**。<br>https://news.powerapple.com/she-hui-qi-wen/2015/3/7/2103371.html or https://archive.is/eKbVX	**Apple Daily, March 7, 2015**<br><br>Jiang Yanyong:<br>**Some of the prisoners were shot, but not yet killed.** They were taken to the hospitals' operating table for the removal of organs, which were then transplanted to the patient. It's an inhuman practice, outrageous.
**Ref. 28**	**2015 年 03 月 06 日 有線新聞台**<br><br>蒋彦永:<br><br>他（北京军区总医院普通外科主任医生李世拥）从来没有肝移植[经验]，他一下就肝移植中心的主任。肝移植病人很多，关键要有供肝。他有办法弄供肝。<br><br>绝大部分中国的肝移植供肝的来源都是枪毙的犯人。那时候没有法律。这个犯人要枪毙了，跟家属通知都不用，同意不同意，一枪毙，肝赶快拿来。<br>…<br>肝移植很重要一条叫做肝脏的热缺血时间愈短愈好。等于打死了，血不流了。如果隔了很长[时间]，这个肝脏功能存活率就低。**后来就打了不完全死，一打，好像死了，马上拉进去，在里头就开始弄肝弄出来**。<br>https://www.facebook.com/icablenews/videos/360349840823757/	**i-Cable News, March 6, 2015**<br>Jiang Yanyong:<br>He (Li Shiyong, director of the department of general surgery of the Beijing Military General Hospital) had no experience in liver transplantation before and became suddenly director of the liver transplantation center. There are many patients waiting for liver transplantation; the key issue is to obtain donor liver. He had his ways to obtain donor livers.<br>The majority of donor livers for transplantation in China were from executed prisoners. At that time, there was no law on this issue. When a prisoner being executed, without informing the family, without asking for their consent, immediately after execution, the liver was taken.<br>It is essential for liver transplantation to keep the warm ischemia period as short as possible. After the execution, the blood stops flowing. If the time is very long, the survival rate of the transplanted liver will be low. **Later, the prisoners were shot, but not completely killed.** [After] one gunshot, [the prisoners] looked like dead. [The bodies] were immediately pulled in, and [the doctors] begun to remove the liver.
